# Self-healing injectable multifunctional hydrogels for intervertebral disc disease

**DOI:** 10.1016/j.mtbio.2025.101655

**Published:** 2025-03-11

**Authors:** Zhengrong Gu, Yi He, Honglin Xiang, Qiwei Qin, Xinna Cao, Ke Jiang, Haoshaqiang Zhang, Yuling Li

**Affiliations:** a Department of Orthopedics, Laboratory of Biological Tissue Engineering and Digital Medicine, Institute of Nanomedicine Innovation and Translational Research, Affiliated Hospital of North Sichuan Medical College, Nanchong, 637000, PR China; b Department of Orthopedics, Affiliated Guang'an District People's Hospital of North Sichuan Medical College, Guang'an County, 638000, PR China; c Department of Orthopedics, Affiliated Nanbu People's Hospital of North Sichuan Medical College, Nanbu County, Nanchong, 637000, PR China; d Department of Orthopedics Surgery, People's Hospital of Xinjiang Uygur Autonomous Region, No. 91, Tianchi Road, Tianshan District, Urumqi, 830001, PR China

**Keywords:** Intervertebral disc degeneration, Hydrogels, Injectable, Self-healing, Bioinformatics

## Abstract

Intervertebral disc degeneration (IVDD) is increasingly prevalent in aging societies and poses a significant health challenge. Due to the limited blood supply to the disc, oral medications and systemic treatments are often ineffective. Consequently, localized injection therapies, which deliver therapeutic agents directly to the degenerated disc, have emerged as more efficient. Self-healing injectable hydrogels are particularly promising due to their potential for minimally invasive delivery, precise implantation, and targeted drug release into hard-to-reach tissue sites, including those requiring prolonged healing. Their dynamic viscoelastic properties accurately replicate the mechanical environment of the natural nucleus pulposus, providing cells with an adaptive biomimetic microenvironment. This review will initially discuss the anatomy and pathophysiology of intervertebral discs, current treatments, and their limitations. Subsequently, we conduct bibliometric analysis to explore the research hotspots and trends in applying injectable hydrogel technology to treat IVDD. It will then explore the promising features of injectable hydrogels in biomedical applications such as drug, protein, cells and gene delivery, tissue engineering and regenerative medicine. We discuss the construction mechanisms of injectable hydrogels via physical interactions, chemical and biological crosslinkers, and discuss the selection of biomaterials and fabrication methods for developing novel hydrogels for IVD tissue engineering. The article concludes with future perspectives on the application of injectable hydrogels in this field.

## Graphical abstract

This review will initially discuss the anatomy and pathophysiology of intervertebral discs, current treatments, and their limitations. It will then explore the promising features of injectable hydrogels in biomedical applications such as drug, protein, cells and gene delivery, as well as tissue engineering and regenerative medicine.

**Figure:**
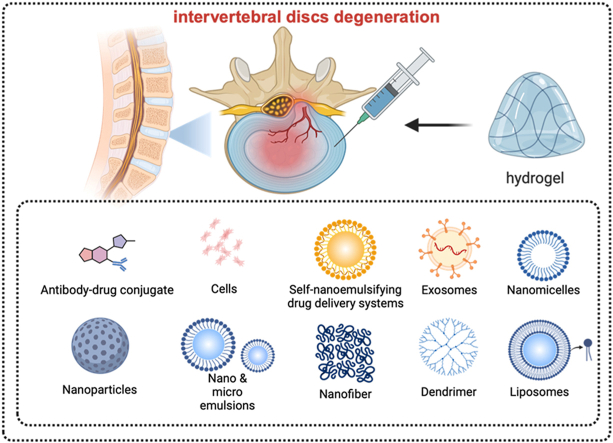

## 1 Introduction

Intervertebral disc degeneration (IVDD) is a common degenerative disease that impacts the quality of life, healthcare systems, and economies globally. Its prevalence is rising, causing significant burden [1,2]. IVDD typically presents as unilateral or bilateral lower back pain, which may be accompanied by stiffness, numbness in the lower limbs, and motor dysfunction. These symptoms can overlap, co-occur, and recur, potentially leading to radiating pain and widespread sensory and motor impairments [3,4].

The intervertebral disc, situated between vertebral bodies, serves as the connecting tissue among spinal vertebrae. It is composed of the central nucleus pulposus (NP), the surrounding annulus fibrosus (AF), and the upper and lower cartilage endplates (CEPs) [5]. The NP, found at the center of the disc, is a soft, gelatinous substance with a high water content, typically between 80 % and 90 %. It primarily consists of chondrocytes and notochordal cells, and features abundant glycosaminoglycans and type II collagen fibers that form a gel-like network. This structure allows the NP to evenly distribute mechanical loads to the surrounding AF, enhancing cushioning performance [6]. The AF, which encases the NP, is primarily made of collagen fibers that help the disc withstand substantial tensile loads and absorb pressure [7]. The CEPs, semi-transparent and homogeneous, mark the boundary between the disc and the vertebral body [8]. They are crucial for transporting nutrients and maintaining fluid pressure within the disc, playing a significant role in its mechanical performance [9]. Degeneration of NP tissue is pivotal in the progression of IVDD [10]. Localized injection therapy has proven more effective than oral medications due to the disc's limited blood supply, making hydrogels an area of intense research focus. Hydrogels, as excellent carriers and ideal substitutes for NP tissues, have become a research hotspot in recent years [11].

Hydrogels, being three-dimensional hydrophilic polymers, are highly biocompatible and emulate normal NP tissue's water absorption and retention characteristics. They are also used as carriers for drugs, proteins, and stem cells, and can be customized for mechanical strength and biological efficacy by modifying their composition and synthesis conditions [12]. This adaptability makes hydrogels valuable in various medical applications, including tissue repair, sustained-release treatments, and functional replacements [13]. Recently, hydrogels have been increasingly studied for treating low back pain caused by IVDD, with some findings already being applied clinically [11]. However, traditional hydrogels have limitations due to their fixed shapes and need for invasive implantation. Additionally, their static network structure restricts cell and nutrient mobility and is vulnerable to external forces, reducing their lifespan in the body. Thus, the development of injectable hydrogels with self-healing properties has garnered interest. These hydrogels can dynamically respond to various conditions—like infrared radiation, UV exposure, temperature, pH, conductive stimulus, or mechanical stress—allowing them to repair and regenerate after damage, similar to the extracellular matrix [14,15]. These hydrogels can break and reform dynamic bonds under specific conditions like infrared radiation, UV exposure, temperature changes, pH variations, conductive stimulus, or shear forces. This ability allows them to self-repair and regain their structure and functionality after damage, mirroring the dynamic, self-healing, and remodeling properties of the extracellular matrix [16,17].

Injectable self-healing hydrogels have emerged as a revolutionary strategy for repairing intervertebral disc degeneration (IVDD), overcoming the static limitations of traditional materials through dynamic reversible crosslinking networks and biomimetic smart-responsive mechanisms (Table 1). Their minimally invasive injectability greatly reduces surgical trauma, while their dynamic viscoelastic properties accurately replicate the mechanical environment of the natural nucleus pulposus, providing cells with an adaptive biomimetic microenvironment in the general development of an analysis of injectable hydrogels with suitable rheological and functional properties for biomedical applications stressing the importance of the viscoelastic properties before and after the injection through the clinical needle, the function injectability and the biphasic material properties through confined compression tests [18]. These hydrogels demonstrate long-term stability under cyclic loading and extended in vivo retention. By integrating pH/MMP-responsive components, gene-cell co-delivery systems, and reactive oxygen species (ROS)-scavenging capabilities, they achieve synergistic therapeutic outcomes that regulate inflammation, alleviate hypoxia, and promote extracellular matrix (ECM) regeneration [19]. This multifunctional design effectively addresses the complex pathological microenvironment of degenerated discs through coordinated mechanical reinforcement and biological repair mechanisms.

**Table 1:** Summary of injectable hydrogels for intervertebral disc regeneration.

Hydrogel Type	Advantages	Disadvantages	Crosslinking Mechanism	References
Chitosan	Binds bioactive molecules; antibacterial; temperature-sensitive	Insoluble in water/organic solvents; needs modification for solubility	pH-sensitive (β-glycerophosphate); chemical (glutaraldehyde)	[[76], [77], [78]]
Alginate	Ca^2+^ crosslinking; oxidation enhances bioactivity; widely used in tissue engineering	High molecular weight limits degradation; lacks protein binding sites	Ionic (Ca^2+^); oxidative (sodium periodate)	[[88], [89], [90]]
Cellulose	Low toxicity; biodegradable; excellent mechanical properties; photocrosslinking mimics IVD	Requires modification for processing	Physical (hydrogen bonding); photopolymerization (methacrylate-modified cellulose)	[70,93]
Hyaluronic Acid	Biocompatible; used in drug delivery and wound healing; gelatin conjugation enhances adhesion	Lacks cell adhesion sites; prone to degradation without crosslinking	Dynamic covalent (Schiff base reaction); physical (hydrogen bonding)	[96,97]
Gelatin	RGD sequences promote adhesion; biodegradable; supports NP-like cell differentiation	Thermosensitive; low mechanical strength without crosslinking	Physical (hydrogen bonding); chemical (glutaraldehyde, genipin)	[106,108,109]
Decellularized ECM	Retains native ECM structure; supports exosome integration	Complex processing; batch variability; degradation rate difficult to control	Thermosensitive self-crosslinking; ionic (Ca^2+^)	[118,119]
Poly(N-isopropylacrylamide)	Temperature-sensitive; good mechanical properties; in situ gelation; bioadhesion enhancement	Reduced mechanical performance at high grafting density; limited cell interactions	Thermoreversible crosslinking (Laponite, RAFT, click chemistry)	[127,128]
Polyethylene Glycol	Hydrophilic and biocompatible; good cell adhesion with modification; biodegradable	Limited cell adhesion without modification; needs additional components for bioactivity	Thiol group-based (4-arm PEG-SH); enzymatic degradation	[[136], [137], [138], [139], [140]]
Polyvinyl Alcohol	Water-soluble; strong; non-toxic; biocompatible	Poor elongation; low fatigue resistance; high friction coefficient	Hydrogen-bonded; dual physical and chemical crosslinking	[144,145]
Poly(lactic-co-glycolic acid)	Biodegradable; good mechanical properties; low immunogenicity	Inflammatory response during degradation; needs co-polymers	Ester bond formation in copolymer structure	[148,149]
pHEMA-co-APMA/PAA	Photopolymerizable; supports MSC differentiation under hypoxic conditions	Requires controlled crosslinking to avoid toxicity	Photocrosslinking (UV polymerization)	[151]

This study outlines the pathology of disc degeneration and reviews current clinical treatments. Then, we employed bibliometric analysis to identify the research direction in applying injectable hydrogel technology for the treatment of IVDD. And discuss various types of injectable hydrogels and the common methods used to manufacture them, highlighting their unique properties and recent research advancements in using these hydrogels to deliver drugs and biologics for treating disc degeneration. It also examines the clinical application, limitations, and challenges of using injectable hydrogels to release therapeutic components at targeted sites, and proposes potential future directions for their development.

## 2 Pathological characteristics of disc degeneration

**Fig. 1:**
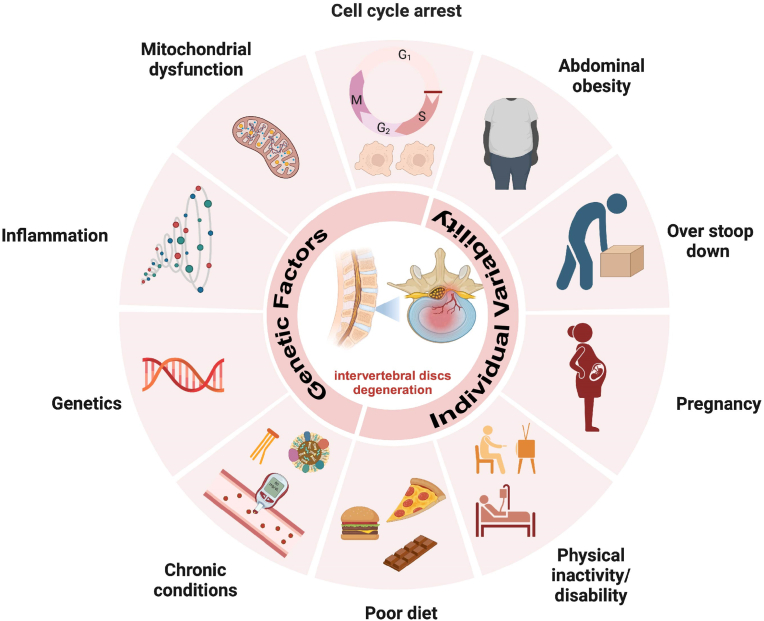
Schematic representation of the main pathogenic factor and pathological changes in IVDD.

The development and progression of IVDD are affected by several factors, including abnormal mechanical loads, stress imbalance, genetic factors, immune system disorders, aging, disrupted nutrient supply, tissue damage, and inflammatory responses [20]. These factors can disturb the disc's homeostasis, leading to IVDD (Fig. 1). During degeneration, there is a decrease in proteoglycan synthesis and a change in collagen synthesis, reducing type II collagen while increasing types I and III [21,22]. There is also an increase in the synthesis and activity of matrix metalloproteinases (MMPs), and calcification of the cartilage endplates reduces nutrient supply, worsening degeneration [23]. Additionally, an imbalance in inflammatory cytokine networks [24], particularly the effects of interleukin-1β (IL-1β) and tumor necrosis factor-α (TNF-α), is considered crucial in causing the pathological changes and pain associated with IVDD [25,26].

Current clinical treatments for IVDD include nucleus pulposus removal, radiofrequency ablation, tissue dissolution, disc replacement, and annulus fibrosus repair [27]. While these treatments may alleviate lower back pain in the short term, they do not support the disc's structural and biomechanical integrity, which is crucial for its function. These treatments disrupt the disc's biomechanical environment and physiological homeostasis [28], and often fail to promote effective regeneration and self-repair of the disc tissues. Consequently, many patients experience postoperative complications, recurring symptoms, or worsened conditions following early minimally invasive treatments [29].

Tissue engineering strategies using biomaterials offer a personalized, less invasive alternative to traditional surgical treatments for degenerative disc disease. These strategies enhance treatment efficacy, slow disease progression, and reduce the risk of surgical complications, thus improving patient outcomes [30]. Injectable treatments, such as small molecules, proteins, genes, platelets, and cells, can target disc inflammation and alleviate pain effectively. However, current injectable treatments suffer from short retention times and half-lives, which limit their long-term effectiveness. Recently, injectable hydrogels have emerged as a promising solution due to their adaptable properties and ability to form into suitable shapes for replacing NP tissue and cushioning spinal stress. These hydrogels can be engineered with various therapeutic agents to maximize their therapeutic potential [31,32].

In this review, we employed bibliometric analysis to identify the research hotspots in applying injectable hydrogel technology to treat IVDD. Based on the objective results of the analysis, we discuss several types of injectable hydrogel systems used in regenerative medicine. We begin by exploring hydrogels formed through physical interactions—such as hydrogen bonds, hydrophobic interactions, host-guest interactions, and ionic interactions—which create a physical network. Next, we examine hydrogels made with non-toxic chemical crosslinkers, discussing reaction mechanisms like Schiff base reactions, Michael addition reactions, and click chemistry involving dialkyne molecules and multi-arm azides. Lastly, we cover hydrogels that use biological crosslinkers, highlighting the use of peroxidases in polymer and hyaluronic acid-tyramine conjugates.

## 3 Analysis of field hotspots based on bibliometrics

Bibliometrics reveals areas and trends of interest to researchers through clustering analysis and visualization of literature [33,34]. This study employed such methods to identify research hotspots and trends in the application of injectable hydrogel technologies for the treatment of IVDD. To obtain relevant literature on the application of injectable hydrogel technologies, we used the Web of Science Core Collection, limiting the language to English. The following advanced search query was applied to extract related literature: TS = ((injectable AND hydrogel) OR “injectable hydrogel”) AND (intervertebral disc OR “spinal disc” OR “intervertebral disc degeneration” OR “spinal disc repair”). A total of 474 documents published between 2014 and 2024 were retrieved. These documents were exported locally in “full record and cited references” and “plain text” formats. After deduplication and removal of retracted papers using CiteSpace 6.4 software, 474 valid documents were included. We further analyzed the retrieved literature's annual publication volume and funding sources. By setting appropriate analytical parameters and selecting a time span from 2014 to 2024, we performed knowledge mapping to examine the co-citation relationships among authors, countries, institutions, journals, and references. Additionally, keyword clustering, burst term analysis, and timeline visualization were conducted to explore collaborations among authors, countries, and institutions and identify key references, research hotspots, and trends in the field.

**Fig. 2:**
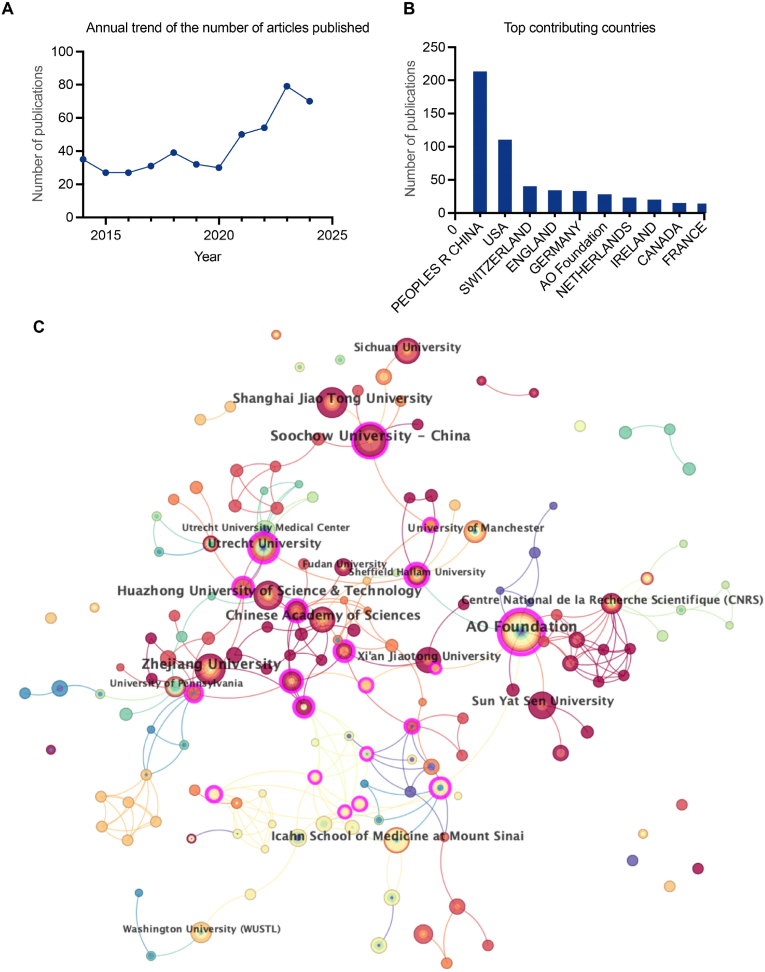
(A). The annual trend in the number of published articles. (B). The distribution of the most contributing countries in the injectable multifunctional hydrogels for intervertebral disc degeneration Research field. (C). The collaboration network among major research institutions in this field.

We first analyzed the annual publication and citation trends related to injectable hydrogels for IVDD and mapped the distribution of contributions by different countries and institutions. Fig. 2A illustrates the annual publication trends from 2015 to 2024, highlighting the growing research interest in this field. Fig. 2B shows the contributions of various countries to this field. China is the largest contributor, with over 200 publications demonstrating its prominent leadership, substantial investments, and significant influence by Chinese research institutions and scholars. The United States ranks second, with over 100 publications showcasing its strong research capabilities and global impact. Switzerland, the United Kingdom, Germany, and the AO Foundation follow, each contributing approximately 30–50 publications. This data indicates a regional distribution of global research, with China and the United States as the primary driving forces behind the field's development.

Fig. 2C presents the collaborative network distribution among major research institutions in the field. Chinese research institutions dominate, particularly Suzhou University, Shanghai Jiao Tong University, Zhejiang University, Huazhong University of Science and Technology, and the Chinese Academy of Sciences. These institutions are represented by larger nodes located at the network's core, signifying high academic output and extensive collaborations. International organizations, such as the AO Foundation, the French National Center for Scientific Research (CNRS), and the Icahn School of Medicine at Mount Sinai, also demonstrate strong academic influence and maintain close collaborations with multiple institutions. The connections between nodes in the figure represent these collaborative relationships. Notably, academic exchange networks have been established between Chinese universities and international institutions, although domestic collaborations remain predominant. Huazhong University of Science and Technology and the Chinese Academy of Sciences, in particular, exhibit remarkable academic influence, serving as pivotal hubs for interinstitutional collaboration.

**Fig. 3:**
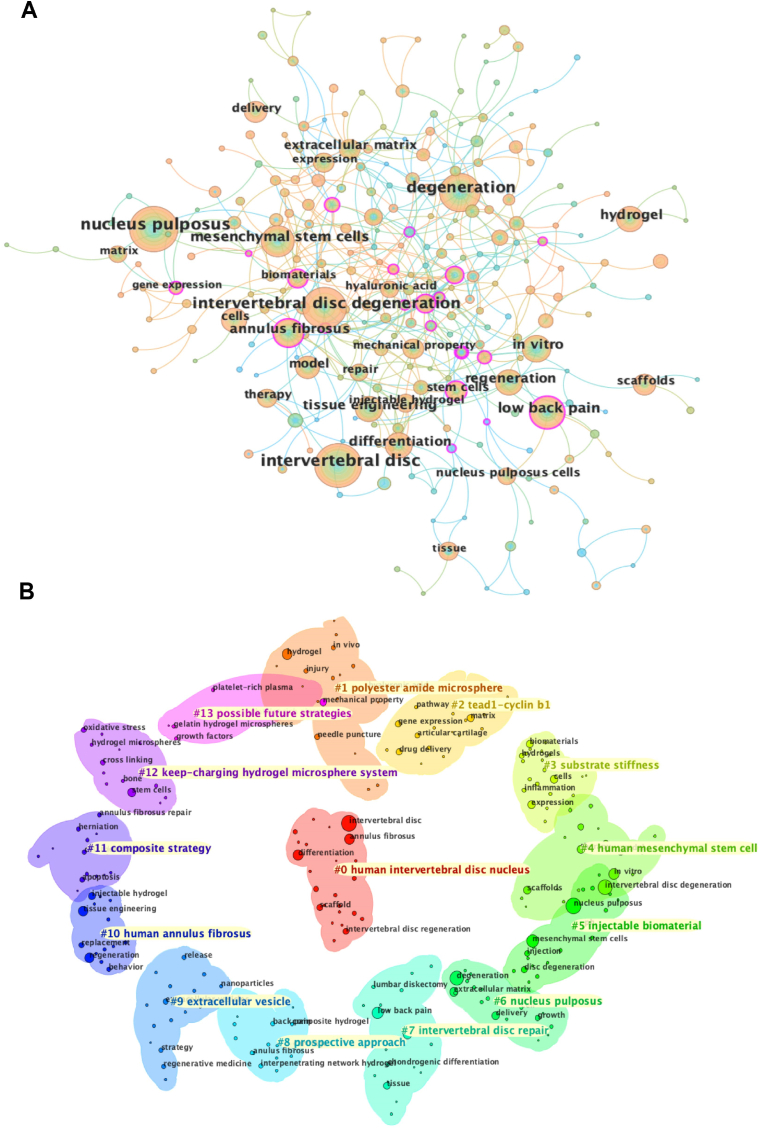
(A). The keyword co-occurrence network map of injectable hydrogels in the treatment of intervertebral disc degeneration. (B). The keyword clustering map of injectable hydrogels in the research field of intervertebral disc degeneration.

Fig. 3A illustrates the keyword co-occurrence network in the field of IVDD, providing insights into research hotspots and thematic distributions identified through CiteSpace analysis. The keyword “intervertebral disc degeneration” occupies the central position, highlighting its role as the primary research focus. Closely associated terms, such as “nucleus pulposus,” “mesenchymal stem cells,” “biomaterials,” and “regeneration,” point to key research themes, including nucleus pulposus regeneration, mesenchymal stem cell-based therapies, and the application of biomaterials. Notably, “hydrogel” emerges as a pivotal keyword, underscoring its importance in the repair of IVDD. It is closely linked to terms such as “scaffolds,” “hyaluronic acid,” and “mechanical property,” reflecting the focus on hydrogels' capacity to enhance mechanical performance and support tissue repair. The term “low back pain” further connects to “therapy” and “tissue engineering,” highlighting the clinical relevance of IVD in managing back pain. Fig. 3B depicts the keyword clustering network, highlighting research trends and emerging directions. Cluster "#0 human intervertebral disc nucleus” represents a focal area, emphasizing studies on nucleus pulposus regeneration and its association with terms like “regeneration,” “annulus fibrosus,” and “scaffolds.” Other notable clusters include "#1 polyester amide microsphere,” highlighting the role of microsphere materials in mechanical enhancement and drug delivery, and "#5 injectable biomaterial,” reflecting the potential of injectable biomaterials. Cluster "#4 human mesenchymal stem cell” underscores the critical role of stem cell-based therapies, while "#9 extracellular vesicle” suggests the growing interest in nanotechnology for tissue repair. Collectively, these clusters focus on intervertebral disc cell regeneration, integrating biomaterials with stem cell therapies, and using hydrogel scaffolds to modulate the degenerative microenvironment. These findings provide a robust foundation for advancing both basic research and clinical applications in IVD therapy.

**Fig. 4:**
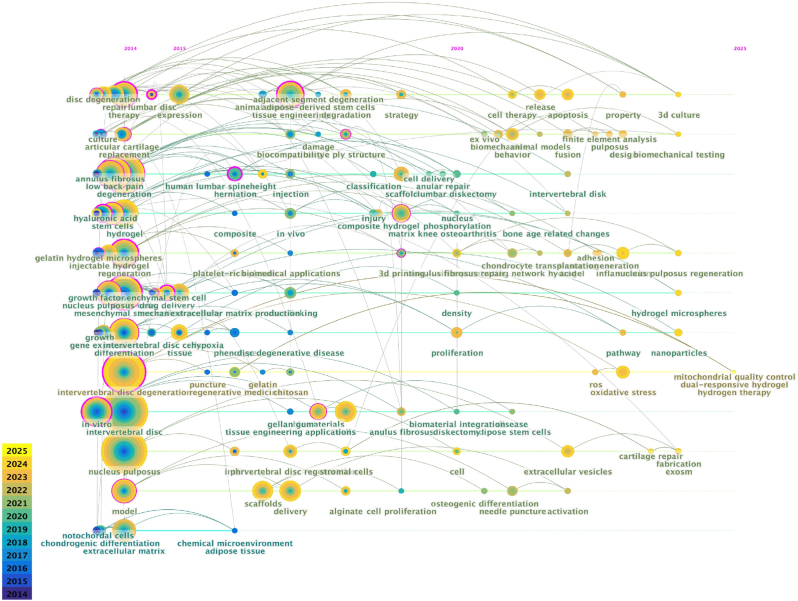
Temporal evolution of research hotspots: a keyword trend analysis.

As illustrated in Fig. 4, it can be seen that research hotspots from 2014 to 2015 mainly focused on fundamental biological issues such as intervertebral disc degeneration. Keywords such as “disc degeneration,” “lumbar disc degeneration,” and “intervertebral disc degeneration” appeared frequently, indicating that research was still primarily concentrated on the mechanisms of disc degeneration and related therapeutic strategies. During this period, studies focused on basic biomedical issues, particularly in the preliminary exploration of stem cells, tissue engineering, and regenerative medicine. From 2016 to 2020, as research deepened, the applications of stem cells and tissue engineering gradually became mainstream in this field. Keywords such as “stem cells,” “mesenchymal stem cells,” and “tissue engineering” appeared with increasing frequency in the chronological diagram, reflecting the significant role of stem cell therapy in intervertebral disc repair. Simultaneously, driven by clinical demands, emerging research areas such as cell delivery, scaffold materials, and biocompatibility further indicated a transition from fundamental research to clinical applications. From 2020 onward, the keywords in the figure show a trend toward greater diversity and interdisciplinarity. For example, emerging technologies such as “hydrogen therapy,” “intelligent hydrogen nanogenerator,” “hydrogel scaffold,” and “3D printing” have become research hotspots. Incorporating these new technologies not only enriches traditional biomedical research but also highlights the integration of biomaterials with intelligent therapeutic strategies, suggesting that future research in this field will increasingly emphasize the combination of technological innovation with clinical practice. Moreover, as time progresses, clinically and application-oriented keywords such as “clinical application” and “mechanical characterization” have gradually assumed greater importance, indicating that the focus of biomedical research has shifted from basic science to practical applications, particularly in treatment strategies for disc diseases, regenerative medicine, and the clinical translation of biomaterials. In summary, the evolution of the chronological diagram demonstrates that the biomedical field has transitioned from basic research to applied research over the past decade, progressively incorporating more innovative and interdisciplinary research directions.

Through CiteSpace analysis, we identified highly cited publications in the field of IVDD (Table 2). Bowles (2017) discussed the application of biomaterials in intervertebral disc repair, garnering 38 citations. Chen (2020) explored the regulation of nucleus pulposus metabolism through gene-hydrogel microenvironments, cited 30 times. Xing (2021) and Clouet (2019) proposed exosome-functionalized hydrogels and bioinspired repair strategies, respectively, with both receiving 25 citations. Similarly, Gan (2017) investigated high-toughness hydrogels for nucleus pulposus regeneration, also cited 25 times. Other notable works, such as Bian (2021) and Schmitz (2020), examined the roles of hydrogels in immune modulation and biomaterial properties. Collectively, these studies highlight the integration of hydrogels and cell-based therapies, advancing technologies for IVD repair.

**Table 2:** Summary of highly cited articles on injectable hydrogels in the field of intervertebral disc degeneration.

Citation Number	Author	Year	Title	Journal	DOI
38	Bowles RD	2017	Biomaterials for intervertebral disc regeneration and repair	*BIOMATERIALS*	10.1016/j.biomaterials.2017.03.013
30	Chen W	2020	Gene‐hydrogel microenvironment regulates extracellular matrix metabolism balance in nucleus pulposus	*ADV SCI*	10.1002/advs.201902099
25	Xing HY	2021	Injectable exosome-functionalized extracellular matrix hydrogel for metabolism balance and pyroptosis regulation in intervertebral disc degeneration	*J NANOBIOTECHNOL*	10.1186/s12951-021-00991-5
25	Clouet J	2019	Intervertebral disc regeneration: from cell therapy to the development of novel bioinspired endogenous repair strategies	*ADV DRUG DELIVER REV*	10.1016/j.addr.2018.04.017
25	Gan YB	2017	An interpenetrating network-strengthened and toughened hydrogel that supports cell-based nucleus pulposus regeneration	*BIOMATERIALS*	10.1016/j.biomaterials.2017.05.017
24	Bian J	2021	Modulation of local overactive inflammation via injectable hydrogel microspheres	*NANO LETT*	10.1021/acs.nanolett.0c04713
23	Schmitz TC	2020	Characterization of biomaterials intended for use in the nucleus pulposus of degenerated intervertebral discs	*ACTA BIOMATER*	10.1016/j.actbio.2020.08.001
23	Binch ALA	2021	Cell-based strategies for IVD repair: clinical progress and translational obstacles	*NAT REV RHEUMATOL*	10.1038/s41584-020-00568-w
23	Knezevic NN	2021	Low back pain	*LANCET*	10.1016/S0140-6736(21)00733-9
20	Gullbrand SE	2017	Translation of an injectable triple-interpenetrating-network hydrogel for intervertebral disc regeneration in a goat model	*ACTA BIOMATER*	10.1016/j.actbio.2017.07.025
20	Sloan SR	2020	Combined nucleus pulposus augmentation and annulus fibrosus repair prevents acute intervertebral disc degeneration after discectomy	*SCI TRANSL MED*	10.1126/scitranslmed.aay2380
20	Bai JY	2020	Reactive Oxygen Species-Scavenging Scaffold with Rapamycin for Treatment of Intervertebral Disk Degeneration	*ADV HEALTHC MATER*	10.1002/adhm.201901186
20	Hartvigsen J	2018	What low back pain is and why we need to pay attention	*LANCET*	10.1016/S0140-6736(18)30480-X

## 4 Physically interacted injectable hydrogels

Physically crosslinked hydrogels leverage non-covalent interactions like hydrogen bonds, hydrophobic interactions, and ionic/electrostatic interactions, which are pivotal in determining their structural and functional properties (Fig. 4). Despite their lower bond energy compared to covalent bonds, these interactions significantly contribute to the mechanical stability of hydrogels. Physical crosslinks typically break first under stress, allowing the hydrogel network to absorb and dissipate mechanical energy effectively. Non-covalent interactions also support the formation of hydrophobic microdomains that enhance the network's stability. Through careful design, these hydrogels can exhibit self-healing, easy processing, and injectability. However, they also face structural instability, lower mechanical strength, and limited durability. The following sections will detail several key physical interactions within hydrogels.

### 4.1 Hydrogen bonds

**Fig. 5:**
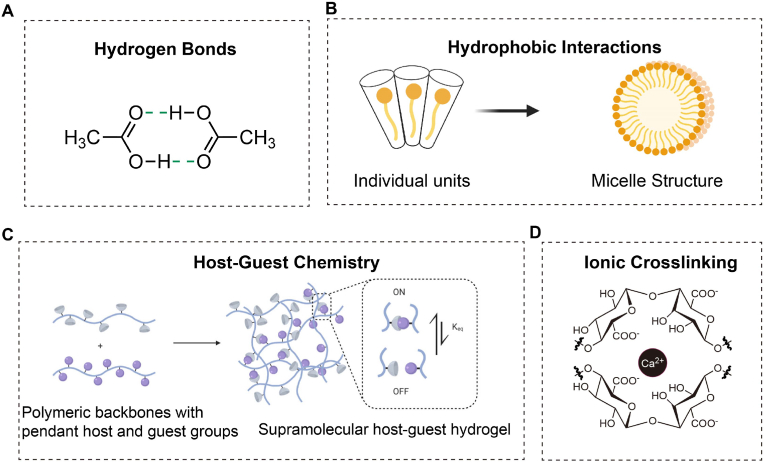
Schematic representation of the non-covalent bonds injectable hydrogels. (A). Hydrogen bondiang, reversible physical crosslinking via hydrogen bonds between molecules; (B). Hydrophobic association, self-assembly of hydrophobic units into micellar structures; (C). Host-guest interaction, supramolecular networks formed by host-guest moieties on polymer chains. Reproduced with permission from Ref. [153]; (D). Ionic interaction, Ionic crosslinking mediated by divalent or multivalent ions (e.g., Ca^2+^) with carboxylate groups on the polymer.

Hydrogen bonds are a prevalent type of non-covalent interaction, usually forming between a hydrogen atom, which carries a partial positive charge, and another atom, such as oxygen, nitrogen, or fluorine [35] (Fig. 5A), which holds a partial negative charge. In a hydrogen bond, the hydrogen atom weakly bonds to one of these electronegative atoms. This interaction occurs because the electron cloud of the hydrogen atom is drawn toward the nuclei of the more electronegative atoms, forming a partial covalent bond. This leads to a temporary polar interaction that enhances intermolecular attraction [36]. Due to their low energy, hydrogen bonds can easily break when temperatures increase, causing hydrogel disintegration. Conversely, the bonds reform when temperatures decrease, allowing the hydrogel to re-gel. Thus, hydrogen bond-based hydrogels can be reversibly controlled by temperature. They also gain injectability and self-healing properties by dynamically crosslinking various polymers through hydrogen bonds. Nevertheless, hydrogen bonds are typically unstable in aqueous environments, and since single hydrogen bonds are weak, multiple hydrogen bonds or a combination with stronger covalent bonds are often employed to enhance the bond strength [37].

### 4.2 Hydrophobic interactions

Hydrophobic interactions occur when hydrophobic surfaces or substances aggregate in aqueous environments. When dispersed in water, hydrophobic polymers cluster together to minimize their contact with water molecules, thus forming a gel network [38] (Fig. 5B). These interactions are generally stronger and more manageable than hydrogen bonds; their strength can be tailored by modifying the shape and concentration of hydrophobic molecules [39]. Hydrophobic interactions are predominantly found in micelles. Amphiphilic polymers and surfactants are added to form these micellar hydrogels, which assemble into micelles through hydrophobic interactions [40]. The sol-gel transition of these polymers is regulated by environmental temperature changes, driven by either the lower critical solution temperature (LCST) or upper critical solution temperature (UCST). Below the LCST, amphiphilic polymers remain soluble, but as the temperature increases, the hydrophobic regions aggregate, forming gel-like structures [41,42].

### 4.3 Host-guest chemistry

Another method to produce injectable physically crosslinked hydrogels is through host-guest chemistry [43] (Fig. 5C). Common host molecules include cyclodextrin (CD), cucurbituril (CB), and crown ether (CE) [44], which can couple with linear polymers or small guest molecules that fit within their cavities [45]. Host-guest interactions utilize the cavities of large-ring molecules to selectively accommodate small molecules through non-covalent bonds like hydrophobic interactions, hydrogen bonds, and π-π stacking [46]. These selective and reversible interactions offer unique advantages in constructing high-strength self-healing hydrogels. For example, CDs with hydrophobic inner cavities can host hydrophobic guest molecules, forming hydrogels that quickly achieve desired mechanical strengths [46]. Reversible hydrogels based on host-guest chemistry are crucial in drug delivery systems, enabling controlled drug release under specific pH or temperature conditions while remaining stable otherwise.

### 4.4 Ionic crosslinking

Ionic crosslinking stabilizes a gel's molecular network through electrostatic interactions between ions (Fig. 5D). In preparing ionically crosslinked hydrogels, solutions or mixtures containing multivalent cations and anions are used. These ions attract each other upon mixing to form ion pairs [47,48] which bind with water molecules to create crosslinking points. This process forms a hydrogel network that captures water molecules, providing stability and elasticity. For example, alginate structures' -COO- groups bond with divalent cations such as Ca^2+^, Cu^2+^, Sr^2+^, and Ba^2+^, forming “egg-box structures” that facilitate controllable ionic crosslinking. These hydrogels exhibit high toughness and good mechanical properties, though their degradation rate may be unpredictable [49]. Additionally, interactions of Fe^3+^ with carboxyl groups in polymer chains can form hydrogels [50]. Changes in pH can affect the degree of ionization, thereby altering the strength of ionic crosslinking. For instance, at low pH, carboxyl groups (-COO^-^) protonate (forming -COOH) reduce crosslinking with metal ions, causing hydrogel disintegration. At high pH, carboxyl groups deprotonate, thereby increasing crosslinking with metal ions, and the hydrogel reforms.

## 5 Chemical bonding

Chemically crosslinked hydrogels generally exhibit greater stability, superior mechanical properties, and adjustable degradation compared to physically crosslinked hydrogels. These are often prepared through dynamic covalent crosslinking mechanisms such as Schiff base reactions, Michael addition reactions, click reactions, and enzymatic processes. While chemical crosslinking offers many benefits, it also has drawbacks, including the need for specific crosslinkers and reaction conditions, which can negatively impact biological tissues.

### 5.1 Schiff base reactions

**Fig. 6:**
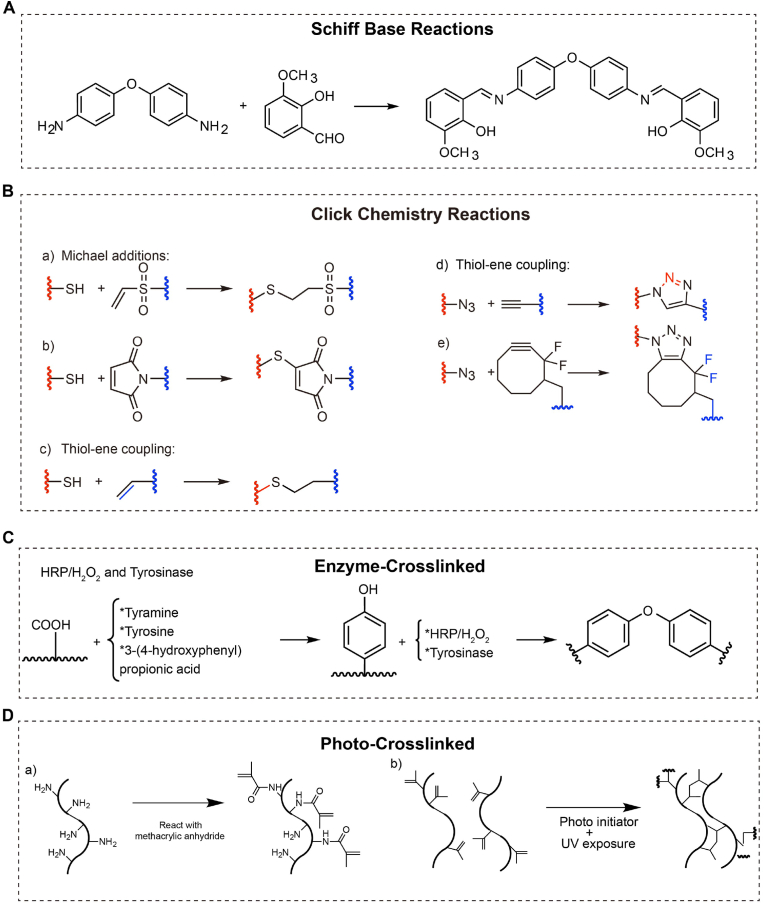
Schematic representation of injectable hydrogels based on the covalent bonds. (A). Schiff Base Reactions, Dynamic covalent imine bonds formed between aldehyde and amine groups; (B). Click Chemistry Reactions, Various bio-orthogonal and highly efficient reactions, including (a, b) Michael additions, (c, d) thiol-ene coupling, and (e) azide-alkyne cycloaddition, facilitate rapid and selective hydrogel formation. Reproduced with permission from Ref. [154]; (C). Enzyme-Crosslinked Injectable Hydrogels, Enzyme-mediated crosslinking using horseradish peroxidase (HRP), hydrogen peroxide (H_2_O_2_), and tyrosinase enables biocompatible hydrogel formation through oxidative coupling of phenolic compounds; (D). Photo-Crosslinked Injectable Hydrogels, initiated by UV exposure and a photoinitiator, induces covalent crosslinking in hydrogels, offering spatiotemporal control over gelation and mechanical properties.

Chemically crosslinked hydrogels are stable, strong, and controllable, maintaining their structure and performance in the challenging microenvironment of IVDD. These hydrogels form covalent bonds between polymer chains using small molecule crosslinkers, which must possess at least two reactive functional groups [51] (Fig. 6A). Schiff bases are a typical form of small molecule crosslinking, created through the condensation of aldehydes with amines [52]. This reaction usually involves compounds with carbonyl (C=O) groups, such as aldehydes or ketones and those with amine (-NH_2_) groups, resulting in imine bonds (C=N). An example is the crosslinking of glutaraldehyde with chitosan amine. Other Schiff base reaction hydrogels include combinations such as dextran/tyramine, hyaluronic acid/tyramine, and periodate-oxidized alginate/gelatin PEG/genipin hydrogels [53,54]. The stability of Schiff base bonds depends on pH; they are stable in neutral or slightly alkaline conditions but break down in acidic environments. This reversibility allows the hydrogel to adapt dynamically in vivo and grants it self-healing properties, as the broken imine bonds can reform under suitable conditions.

### 5.2 Click chemistry reactions

Click chemistry reactions excel over traditional chemical crosslinking methods due to their rapid reaction rates, high efficiency, selectivity, minimal side reactions, and low cytotoxicity. These attributes make them extremely useful in biomedical engineering. Key types of click chemistry include Michael addition, thiol-ene reactions, azide-alkyne cycloaddition, and Diels-Alder cycloaddition. Michael addition involves introducing nucleophilic compounds [55,56] to crosslink or functionalize hydrogels, utilizing reactants such as donors (thiols, amines) and acrylates and vinyl sulfone as acceptors (acrylate, acrylamide, vinyl sulfone, maleimide) [57] (Fig. 6B). Thiol-ene reactions, also known as thiol-ene additions, form thioether products through reactions between thiols (–SH functional groups) and alkenes (carbon-carbon double bonds) [58]. However, the azide-alkyne cycloaddition's use of cytotoxic copper catalysts restricts its biomedical applications [59,60]. Additionally, nucleophilic ring-opening reactions and Diels-Alder cycloaddition, particularly 1,3-dipolar reactions involving copper ions, extend the scope of click chemistry (heterocyclic Diels-Alder reactions) [61]. These reactions are instrumental in designing injectable hydrogels for targeted release and improved hydrogel properties, with the specific method chosen based on the required properties and applications holding significant potential in precise therapy and tissue engineering.

### 5.3 Enzyme-crosslinked injectable hydrogels

Enzyme-based hydrogels are formed through enzyme-catalyzed reactions specific to the polymers used [62] (Fig. 6C), under mild conditions compatible with cellular environments, such as neutral pH and physiological temperatures. The enzymatic process does not affect the catalyst itself. Since enzymes are sensitive to environmental conditions like pH and temperature, these factors must be carefully managed to prevent deactivation [63]. Enzymatic crosslinking is highly specific, reacting only with designated polymolecules without inducing cytotoxic effects [64]. This method facilitates rapid gelation, which is beneficial for maintaining hydrogel structure and preventing cell sedimentation during formation. For instance, hydrogels created by horseradish peroxidase (HRP) involve the enzyme catalyzing the crosslinking of polymer-phenol conjugates in the presence of hydrogen peroxide, allowing for control over gelation rates and densities [65].

### 5.4 Photo-crosslinked injectable hydrogels

Photo-crosslinked hydrogels are typically made by introducing molecules with photopolymerizable double bonds, such as ethylene, acrylic acid, acrylamide, and acrylates. These hydrogels are often derived from natural polymers modified with acrylate or acrylamide derivatives, achieved by acylating natural polymers with active groups (Fig. 6D). When photoinitiators are present, these hydrogels can form under mild conditions [66]. Photo-crosslinked hydrogels offer advantages such as fast reaction rates, easily controllable shapes, and minimal heat release during reactions. However, the synthetic monomers and photoinitiators are generally toxic, posing challenges in removing harmful substances and radical residues. Future research should focus on developing chemically modified natural materials that are safer and meet practical requirements [67].

## 6 Injectable natural hydrogels

**Fig. 7:**
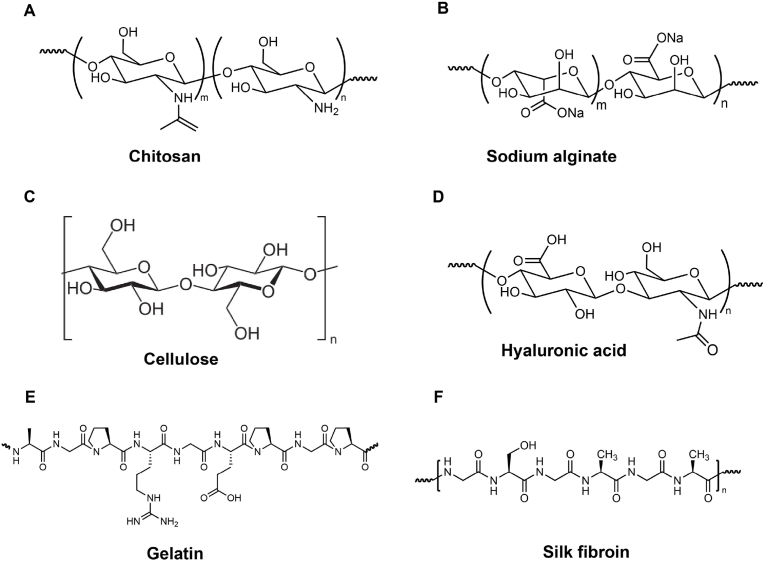
Schematic structure of injectable hydrogels based on natural source. (A) Chitosan; (B) Sodium Alginate; (C) Cellulose; (D) Hyaluronic Acid; (E) Gelatin; (F) Silk Fibroin.

Ideal hydrogels for intervertebral disc (IVD) regeneration should have characteristics like excellent biocompatibility and biodegradability, mechanical properties that mimic the natural nucleus pulposus, injectability for conservative or minimally invasive surgery, and immediate solidification upon injection to prevent leakage and protect adjacent nerves and blood vessels. Hydrogels are classified based on their polymer sources into three types: natural (e.g., alginate, chitosan, cellulose, gelatin, and hyaluronic acid) (Fig. 7), synthetic (e.g., polyethylene glycol, polyurethane, and polyvinyl alcohol) (Fig. 9), and composite.

**Fig. 8:**
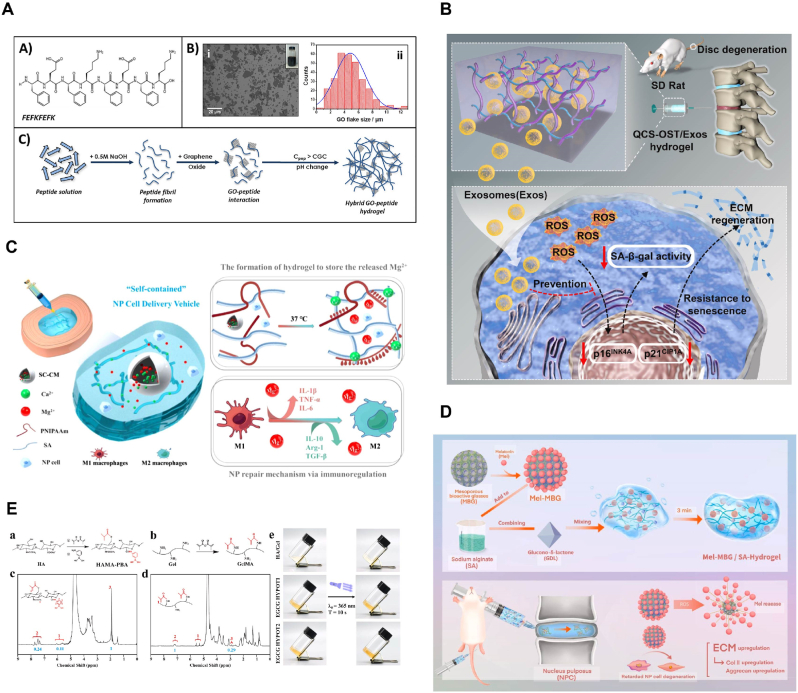
Schematic of injectable hydrogels based on natural source. (A). Chemical structure of FEFKFEFK peptide and Schematic representation of the formulation route used to prepare peptide/GO hybrid hydrogels. Reproduced with permission from Ref. [71]. (B). Schematic illustration of the QCS-OST/Exos hydrogel promoting disc regeneration. Reproduced with permission from Ref. [72]. (C). Schematic showing the composition of the injectable self-contained SA/PNIPAAm hydrogel, its working principle and functionality. Reproduced with permission from Ref. [73]. (D). Schematic diagram for the preparation of injectable MBG-Mel/SA hydrogel. Reproduced with permission from Ref. [74]. (E). Design and formation of HAMA-PBA, GelMA and EGCG HYPOT. Reproduced with permission from Ref. [75].

Natural hydrogels from natural polymers degrade into monosaccharides or disaccharides under specific biological conditions or enzymatic catalysis. Their degradation products are biocompatible, absorbable, and can provide energy to organisms [68]. These properties make natural hydrogels safe, non-toxic, and ideal for biomedical applications due to their high water retention, renewability, biodegradability, and biocompatibility [69]. The differences in properties among various natural polysaccharides arise from variations in functional groups and polymer chain lengths. Recent research has explored developing injectable hydrogels from chitosan, sodium alginate, cellulose, and hyaluronic acid for treating degenerative disc diseases [[70], [71], [72], [73], [74], [75]] (Fig. 8A–E).

**Fig. 9:**
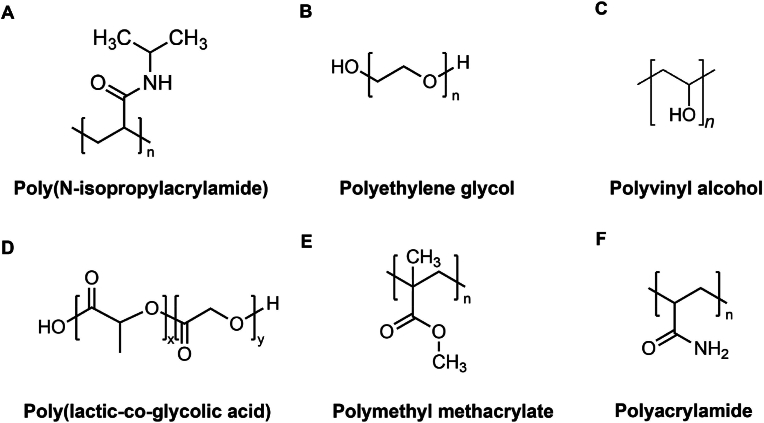
Schematic structure of injectable hydrogels based on synthetic biomaterials. (A) Poly(N-isopropylacrylamide); (B) Polyethylene glycol; (C) Polyvinyl alcohol; (D) Poly(lactic-co-glycolic acid); (E) Polymethyl methacrylate; (F) Polyacrylamide.

### 6.1 Based on chitosan

Chitosan, derived from the deacetylation of chitin from marine crustaceans, is insoluble in water and most organic solvents due to its crystalline regions and hydrogen bonds, dissolving only in dilute acids [[76], [77], [78]]. To improve solubility, functional groups are introduced at its amino and hydroxyl positions. Chitosan is positively charged, making it suitable for carrying negatively charged bioactive molecules and has antibacterial properties [79]. When combined with β-glycerophosphate (C/GP), chitosan forms a temperature-sensitive injectable hydrogel that gels around 37 °C [80].

Temperature-sensitive composite chitosan-based hydrogels have shown promise in repairing disc defects. Lachlan J. Smit et al. [81]. Developed an injectable hydrogel composed of dextran, chitosan, and bone minerals for NP regeneration, retaining its integrity under cyclical loading. Li et al. [82] created a thermosensitive injectable hydrogel based on N-hexanoylation of glycol chitosan for treating degenerative disc disease, stable for over 28 days in an ex vivo porcine model, and enhancing celecoxib release [83,84]. Yasaman Alinejad et al. [85] studied thermosensitive chitosan hydrogels prepared with sodium bicarbonate, β-glycerophosphate, and phosphate-buffered saline. Wang et al. [86] designed a pH-responsive injectable hydrogel for miRNA delivery, composed of glycidyl methacrylate-modified carboxymethyl chitosan and assembled through an amino-alkyne click reaction, suitable for IVDD repair. Additionally, Guan et al. [87] developed a quaternized chitosan and oxidized starch-based hydrogel for disc degeneration treatment, promoting ECM remodeling and partially restoring NP and annulus fibrosus structures in a rat model.

### 6.2 Based on alginate

Alginate, a hydrophilic polysaccharide polymer extracted from brown algae, consists of α-L-guluronic acid and β-D-mannuronic acid linked by β-1,4-glycosidic bonds. Sodium alginate forms hydrogels readily with divalent ions, particularly calcium [88]. Alginate hydrogels are created via external gelation (mixing sodium alginate with Ca^2+^ ions) or internal gelation (using calcium carbonate and glucono-δ-lactone). High molecular weight alginate is difficult to degrade and lacks binding sites for amino acids and proteins, limiting in vivo applications. Structural modifications, such as oxidation with sodium periodate to introduce reactive aldehyde groups and RGD peptide introduction, have expanded its tissue engineering uses [89,90]. Christopher J. Panebianco et al. [91] designed an injectable composite biomaterial using oxidized alginate microbeads to deliver annulus fibrosus cells within a high-modulus fibrinogen hydrogel crosslinked with genipin. This composite stabilized intervertebral discs and protected cells essential for long-term healing, showing better mechanical and biological repair in a bovine caudal intervertebral disc culture bioreactor. Feng et al. [92] combined mesoporous bioactive glass nanoparticles with melatonin, ultrasonically processed for drug loading, into a sodium alginate solution. Injected into rat tail nucleus pulposus, this solution solidified in situ, releasing calcium ions and providing effective repair.

### 6.3 Cellulose

Cellulose is a linear natural polymer, formed by β(1 → 4)-linked d-glucose units and is abundant in plants and bacteria. It is the most plentiful natural polysaccharide, noted for its low toxicity, ease of processing, biodegradability, and renewability [70]. Its many hydroxyl groups allow for the formation of intermolecular hydrogen bonds, providing excellent chemical stability and mechanical properties suitable for tissue engineering scaffolds [93]. D.M. Varma et al. [94] developed a novel hydrogel from carboxymethyl cellulose and methylcellulose, which gels upon exposure to light due to methacrylate-modified cellulose polymers. This hydrogel replicates the mechanical properties of natural nucleus pulposus tissue, potentially improving spinal disc mechanics and durability under severe stress. Schmocker et al. [95] reported a new photocrosslinkable polyethylene glycol dimethacrylate and nanofibrillated cellulose composite hydrogel tailored to natural tissue properties. Implanted into bovine discs via a minimally invasive device, this hydrogel maintained disc height even after extensive loading cycles. However, the broader application of such photocrosslinkable hydrogels is often limited by the specific requirements for the crosslinking equipment and environment.

### 6.4 Based on hyaluronic acid

Hyaluronic acid (HA) is a connective tissue polysaccharide made of D-glucuronic acid and N-acetyl-D-glucosamine, used in drug delivery and wound dressing applications [96]. It is produced by hyaluronan synthase on the plasma membrane and secreted into the ECM [97]. For early-stage IVDD treatment, encapsulating stem cells or nucleus pulposus cells in hydrogels is common. However, HA hydrogels lack cell adhesion sites. To address this, Chen et al. [98] developed an injectable hydrogel using a Schiff base reaction between oxidized hyaluronic acid and gelatin-adipic dihydrazide (oxi-HAG-ADH), enhancing cell adhesion with high-molecular-weight (1900 kDa) HA. The hydrogel's viscoelastic properties matched natural nucleus pulposus tissue, with a similar complex shear modulus (11–14 kPa vs. 11.3 kPa). Jessica E. Frith et al. [99] also created an injectable hydrogel with enzyme-crosslinked polyethylene glycol and HA, enhanced with pentosan polysulfate (PPS), promoting chondrogenic differentiation and collagen type II deposition. A novel 2.6 % H-HA/3.2 % sorbitol combination containing 52 mg of stabilized high molecular weight (H-HA) pure hyaluronic acid and 64 mg of sorbitol in phosphate buffer solution was recently developed [100]. The elastic modulus G′ reaches 529 Pa and the viscosity η reaches 856 Pa-s. The hyaluronic acid chains within the gel are connected not by chemical covalent bonds created by crosslinking agents, but by hydrogen bonds, which are low-energy physical bonds that can be easily broken and restored depending on the mechanical action applied to the gel product. The unique rheological properties achieve uniform distribution, providing mechanical support while stimulating tissue regeneration.

Conditional EGFR deletion in mice increased neuropeptides, ECM, and autophagy markers [101]. Gefitinib, an FDA-approved EGFR inhibitor, enhances ECM production rich in collagen type II in IVD by inhibiting EGFR [102,103]. Pan et al. [101] applied a thermosensitive injectable hyaluronic acid hydrogel, AHA-g-PNIPAAm, for gefitinib delivery, controlling its release to restore degenerative IVDs. Luo et al. [104] designed an injectable self-antioxidant hydrogel (HA/CS) with improved anti-inflammatory performance, delivering chondroitin sulfate (CS) to treat IVDD, featuring rapid formation, excellent injectability, mechanical properties, and pH-responsive behavior. Yang et al. [105] developed an injectable hydrogel, PBNPs@OBG, with antibacterial, antioxidant, rapid gelation, self-healing properties, and dual dynamic bond crosslinking. An EGCG hydrogel library (EGCG HYPOT) was created using borate ester reactions between EGCG and phenylboronic acid (PBA), offering anti-inflammatory and antioxidant effects with injectability, shape adaptability, and efficient EGCG loading, showing good mechanical properties, tissue adhesion, and sustained acid-responsive EGCG release [75].

### 6.5 Gelatin

Gelatin (Gel), derived from collagen, forms gels in water through hydrogen bonding, enhancing cell adhesion and growth via the Arg-Gly-Asp (RGD) sequence. Wang et al. [106] developed a nanostructured gelatin colloidal hydrogel with mesenchymal stem cells (MSCs) for treating intervertebral disc (IVD) degeneration. This hydrogel mimics natural nucleus pulposus (NP) properties, showing biocompatibility, biodegradability, and supporting NP-like MSC differentiation. Another study created a type II collagen/chondroitin sulfate (CS) composite hydrogel with genipin for adipose-derived stem cell (ADSC) delivery. Xu et al. [107] introduced an injectable gefitinib-crosslinked chitosan/type I collagen hydrogel (Ge-CS/COL-I), suitable for NP tissue engineering and showing significant NP structure restoration in a rat model after 8 weeks. Ferulic acid (FA) was used in an injectable chitosan/gelatin/glycerophosphate (C/G/GP) hydrogel to combat oxidative stress in NP cells caused by hydrogen peroxide (H_2_O_2_), effectively mitigating stress [108,109]. Luo et al. synthesized an injectable bioorthogonal hydrogel (BIOGEL) using tetrazine-norbornene ligation, solidifying in 5–10 min post-injection. In a rat model, BIOGEL containing TGF-β improved tissue structure, matrix synthesis, and functional recovery, showing potential for various degenerative conditions [110].

### 6.6 Others

Injectable hydrogels from alginate, chitosan, cellulose, gelatin, and hyaluronic acid mimic natural ECM and provide cell scaffolds [111]. NP-derived mesenchymal stem cells (NPMSCs) show high tolerance to harsh microenvironments and effectively regenerate degenerated NP tissues in rat models [[112], [113], [114]] Peptide-modified GAG hydrogels can self-assemble rapidly in situ, restoring the mechanical properties of nucleotomized discs within seconds to minutes [115]. Chen et al. [116] designed the OG/GCA hydrogel, crosslinked with dynamic bonds like acylhydrazone and imine bonds, and embedded with therapeutic siRNA for precise gene release in acidic and inflammatory environments. This hydrogel maintains gene-drug release for over 28 days, reducing inflammatory factors and enhancing IVD regeneration by inhibiting the P65/NLRP3 signaling pathway [116]. Adrián Pérez-San Vicente et al. [117] developed a self-healing hydrogel based on gold(I)-thiolate/disulfide exchange, which restores the biomechanical properties of nucleotomized IVDs to those of intact IVDs, including a stiffening effect at increasing frequencies.

The extracellular matrix (ECM) is a complex network that provides structural support, signaling, and cellular interactions. Decellularized ECM (dECM), created by removing cells, maintains the original matrix structure and function [118,119]. Xing et al. [120] developed a thermosensitive dECM hydrogel combined with adipose-derived mesenchymal stem cell (ADSC) exosomes (dECM@exo). This hydrogel promotes in situ gelation, NPC growth, and matrix synthesis, while reducing inflammation and cell apoptosis. Animal studies showed that dECM@exo maintains IVD microenvironment homeostasis and improves IVDD. Chiara Borrelli et al. [104] created an injectable biomimetic material combining dECM from bovine NP tissue with functionalized chitosan (CS), enhancing NP cell morphology and matrix deposition.

The customized multiphase nucleus/annulus scaffold strategy provides a new research direction for IVD tissue engineering [121]. Studies have shown that an annular scaffold composed of poly(ε-caprolactone) (PCL) with precisely designed structures can effectively mimic the characteristics of the native annulus fibrosus. Meanwhile, the nucleus material consists of a cell-loaded collagen–low molecular weight hyaluronic acid-based hydrogel, which possesses specific rheological and functional properties. Rheological tests indicate that this hydrogel exhibits shear-thinning behavior, allowing smooth injection into the nucleus and ensuring effective filling. Comprehensive analysis of the scaffold demonstrates that its viscoelasticity and mechanical properties are well-suited for IVD repair applications. Furthermore, mechanical characterization results confirm that the scaffold's compressive modulus falls within the physiological range of the lumbar intervertebral disc and exhibits a typical J-shaped initial stress-strain curve, characteristic of native IVD, indicating excellent mechanical compatibility.

## 7 Injectable hydrogels based on synthetic biomaterials

**Fig. 10:**
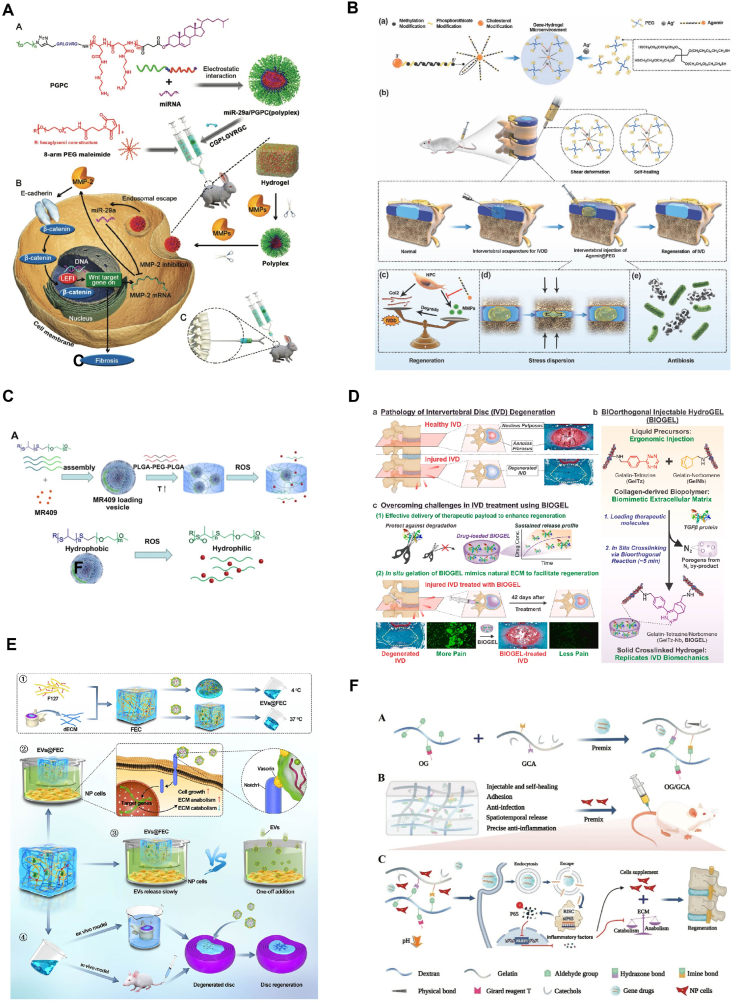
Schematic of injectable hydrogels based on synthetic biomaterials. (A). Schematic illustration for formation of miRNA/PGPC polyplex micelles [122]. (B). Schematic illustration of The Agomir@PEG Gene-hydrogel microenvironment for regeneration of IVDD. Reproduced with permission from Ref. [123]. (C). Schematic illustration of the thermosensitive hydrogel loaded with ROS-responsive PPS-PEG vesicles for controlled release of MR409. Reproduced with permission from Ref. [124]. (D). Schematic for injectable bioorthogonal hydrogel (BIOGEL) for intervertebral disc (IVD) regeneration. Reproduced with permission from Ref. [110]. (E). Diagrammatic sketch of FEC hydrogel fabrication and its utilization in intervertebral disc regeneration. Reproduced with permission from Ref. [125]. (F). Schematic diagram of the synthetic route for the gene drug-loaded multifunctional hydrogel and its gene-cell combination therapy application for IVD regeneration precisely targeting the P65/NLRP3 signal pathway. Reproduced with permission from Ref. [126].

Injectable hydrogels based on synthetic biomaterials can be delivered in liquid form and solidify at the target site. Comprising synthetic polymers like polyethylene glycol (PEG), poly(N-isopropylacrylamide) (PNIPAAm), polyvinyl alcohol (PVA), polyethyleneimine (PEI), and polyacrylic acid (PAA) (Fig. 9), these hydrogels offer customizable mechanical, chemical, and biological properties. The synthesis process allows precise control, consistency, and stability, making these hydrogels ideal for tissue engineering applications [110,[122], [123], [124], [125], [126]]. (Fig. 10A–F).

### 7.1 Poly(N-isopropylacrylamide) (pNIPAM)

Poly(N-isopropylacrylamide) (PNIPAAm) is a temperature-sensitive polymer that transitions to a gel state near 32 °C [127]. Its temperature sensitivity stems from hydrophilic amide bonds and hydrophobic isopropyl groups, transitioning from soluble at lower temperatures to a dense globular structure at LCST. PNIPAAm-grafted chondroitin sulfate (PNIPAAm-g-CS) hydrogels, which form in situ, exhibit enhanced mechanical properties and bioadhesion strength at 37 °C, suitable for NP regeneration [128]. An injectable, LAPONITE-crosslinked PNIPAAm-co-DMAc hydrogel promotes the differentiation of BMPCs into NP-like cells without additional growth factors [129]. Malonzo et al. created a thermo-reversible PNIPAAm hydrogel that remains liquid at room temperature and solidifies above 32 °C [130]. An injectable PNIPAAm-based hydrogel delivering SHP099 targets SHP2 to repair degenerated IVD [131]. A self-sustaining hydrogel with sodium alginate, PNIPAAm, and silicate ceramics, which solidifies at body temperature, promotes local cell matrix synthesis and immunomodulation [132].

Mortisen et al. synthesized thermoreversible HA-PNIPAM hydrogels using RAFT polymerization and click chemistry, optimizing properties for cell therapy [133]. They found that increasing PNIPAM levels decreased viscosity at 20 °C and increased the elastic modulus above 30 °C, although higher grafting densities reduced mechanical performance. These hydrogels improved hMSC differentiation into a disc phenotype in vitro [134]. Marianna Peroglio developed thermoreversible hydrogels by grafting PNIPAM onto a hyaluronic acid backbone, promoting NP cell redifferentiation and activating endogenous cells in the disc [135].

### 7.2 Polyethylene glycol (PEG)

Polyethylene glycol (PEG) derivatives are essential for creating injectable hydrogels due to their hydrophilicity and biocompatibility, reducing immune system recognition [136,137]. Although PEG hydrogels mimic nucleus pulposus (NP) tissue properties, their lack of cell adhesion limits their regenerative use. Combining PEG with components like PLA, RGD sequences, or enzyme-sensitive peptides enhances cell adhesion and proliferation [[138], [139], [140]]. Agomir874-loaded PEG hydrogel, designed to suppress matrix metalloproteinase (MMP) expression, was developed using 4-arm PEG-SH and silver ion solutions, featuring self-healing and antibacterial properties. This hydrogel balances ECM synthesis/degradation and enhances tissue regeneration in degenerative discs [123]. Aubrey et al. created an injectable laminin-111 functionalized PEG (PEG-LM111) hydrogel, which significantly improved NP cell retention over 14 days [141,142]. Huang et al. reported a chitosan/PEG hydrogel (CSMA-PEGDA-L) with superior compressive strength and low cytotoxicity, slowing IVD degeneration progression in rat models. An interpenetrating polymer network (IPN) hydrogel with dextran, gelatin, and PEG supported NP cell proliferation and matrix deposition, promoting NP tissue regeneration in pigs [143]. An injectable MMP-degradable hydrogel with miR-29a encapsulated micelles was developed for sustained, bioresponsive delivery to NP cells, effectively silencing MMP-2 expression and reversing IVDD in animal models [122].

### 7.3 Polyvinyl alcohol (PVA)

Polyvinyl alcohol (PVA) is a water-soluble polymer synthesized from vinyl acetate through polymerization and hydrolysis. Known for its water solubility, tissue compatibility, safety, non-toxicity, and skin non-irritation, PVA forms hydrogels via hydrogen bond-mediated crosslinking, creating non-toxic hydrogels with high mechanical strength. These hydrogels resemble human intervertebral disc tissue and are used in injectable artificial disc replacement procedures [144]. To develop an in situ forming 3D network that retains injectability, Gemma et al. combined PVA with polyvinylpyrrolidone, which exhibited good viscoelastic behavior under dynamic shear and compression. The thixotropic nature of the composite hydrogel ensured good injectability, making it promising for targeted drug delivery in spinal diseases [145]. Yener et al. developed a novel hydrogel scaffold loaded with insulin-like growth factor 1 (IGF-1) and bone morphogenetic protein-2 (BMP-2) using borax and PVA chitosan/starch [146]. This hydrogel facilitated controlled growth factor release, enhancing proliferation and extracellular matrix secretion in annulus fibrosus and nucleus pulposus cell cultures, making it suitable for disc drug delivery. Despite its high water content and good mechanical properties, PVA hydrogels have limitations such as poor elongation at break, fatigue resistance, and high friction coefficient. Li et al. improved PVA hydrogels through physical and chemical dual crosslinking with formaldehyde, forming stronger acetal linkages and addressing mechanical deficiencies. The enhanced hydrogel demonstrated over 600 % elongation, a 42 % reduction in modulus loss after fatigue testing, and a significantly reduced average friction coefficient, meeting clinical needs for applications like meniscus, cartilage, and intervertebral discs. However, challenges such as residual crosslinking agents and poor cell compatibility remain [147].

### 7.4 Poly(lactic-co-glycolic acid) (PLGA)

Poly(lactic-co-glycolic acid) (PLGA) is a biodegradable copolymer of lactic and glycolic acids. Its metabolites can be excreted through the tricarboxylic acid cycle, ensuring good biocompatibility without significant inflammation or rejection reactions. PLGA can be combined with various polymers to form hydrogels for different applications. Bevacizumab, a vascular endothelial growth factor (VEGF) inhibitor, has shown potential in treating IVDD through local drug delivery. High VEGF expression in degenerated human and rat intervertebral discs has been observed, and bevacizumab treatment has led to reduced MMP3 expression and increased collagen II synthesis, improving disc degeneration. Injectable thermosensitive PLGA-PEG-PLGA hydrogels are being explored as a treatment for disc degeneration in chronic low back pain patients [148]. Additionally, Cheng et al. designed a novel injectable composite hydrogel scaffold: an oligonucleotide [poly(ethylene glycol) fumarate]/sodium methacrylate (OPF/SMA) hydrogel scaffold loaded with dual-drug/release PLGA microspheres containing IL-4 and kartogenin (KGN). This scaffold has good mechanical properties and low immunogenicity while promoting sustained drug release [149]. The IL-4-PLGA microspheres help induce macrophage polarization from the M1 to M2 phenotype during early induction and demonstrate sustained anti-inflammatory effects through KGN-PLGA microspheres.

### 7.5 Others

Synthetic polymers typically exhibit high mechanical strength and stability, while natural polymers are known for their superior biocompatibility and bioactivity. Merging these two types can create hydrogels that leverage synergistic benefits. This study introduces a method to prepare injectable silk fibroin/polyurethane (SF/PU) composite hydrogels via chemical crosslinking under physiological conditions [150]. Hu et al. developed an SF/PU composite hydrogel that can be administered through a small incision. Bone marrow stem cell (BMSC) proliferation assays showed positive cell viability and significant growth over seven days. Additionally, in situ, photopolymerizable injectable synthetic hydrogels encapsulating human mesenchymal stem cells (hMSCs) are promising options for nucleus pulposus (NP) tissue repair and regeneration. A novel photocrosslinkable, injectable synthetic polymer hydrogel (pHEMA-co-APMA grafted with polyamide amine (PAA)) has proven effective in encapsulating and differentiating hMSCs into NP phenotypes under hypoxic conditions, indicating its potential for restoring NP tissue function and mechanical properties [151]. Tellegen et al. [152] reported a PCLA-PEG-PCLA hydrogel that responds to temperature changes within a specific range (e.g., near body temperature), forming gel. This temperature responsiveness primarily stems from the characteristics of PEG and can be adjusted by altering the molecular weight and concentration of PEG. The PCLA-PEG-PCLA hydrogel has shown biocompatibility and practicality for intradiscal applications. Injecting a celecoxib-loaded hydrogel into canine intervertebral discs (IVDs) experiencing early spontaneous degeneration significantly alleviated back pain in nine out of ten dogs, demonstrating the potential of this hydrogel to evolve as a new therapeutic biomaterial for IVD degeneration.

## 8 Summary

Injectable hydrogels are promising scaffolds for intervertebral disc (IVD) tissue engineering, owing to their minimally invasive application and ability to adapt to irregular defects. This review summarizes common crosslinking techniques for hydrogel synthesis involving physical and chemical strategies and explains their specific mechanisms and conditions for hydrogel formation. Physical and chemical crosslinking methods are highlighted, noting that non-covalently crosslinked hydrogels offer reversibility, biomimicry, bioactivity, and biodegradability advantages. However, these hydrogels often lack structural stability, exhibit lower mechanical strength, and have limited durability. Conversely, hydrogels synthesized through chemical methods provide robust stability and excellent mechanical performance under physiological conditions, though in vivo chemical reactions may pose risks. Injectable hydrogels are characterized by their ability to reshape and self-heal, adapt their structure under external forces, maintain new shapes, or return to their original forms upon force removal. They can repair their structure and functionality after physical or chemical damage through interactions like hydrogen bonding, van der Waals forces, ionic bonds, and Schiff base bonds, showcasing remarkable adaptability and functionality in the dynamic in vivo environment.

This review covers the mechanisms of dynamic, self-healing injectable hydrogels and summarizes their applications in treating intervertebral disc (IVD) diseases using natural and synthetic biomaterials. Natural biomaterials like chitosan, collagen, gelatin, alginate, fibrin, elastin, heparin, and hyaluronic acid are preferred for their excellent cell compatibility, biodegradability, low toxicity, and tissue similarity. However, their mechanical strength is often inadequate, limiting their use. In contrast, synthetic biomaterial-based hydrogels provide better stability and mechanical properties but lack optimal biocompatibility and bioactivity. Despite advances in using hydrogels for IVD degeneration, several challenges persist.

(1) Key considerations include balancing self-healing capabilities, injectability, and physical stability. Injectable hydrogels must possess the right rheological properties for quick, stable gel formation in vivo, which minimizes leakage and maximizes therapeutic outcomes.
(2) It is also essential to ensure the biocompatibility of hydrogels with IVD tissues to prevent immune reactions and other complications.
(3) Mechanical Stability and Support Post-Gelation: After injection, the hydrogels should mimic the natural mechanical support of the nucleus pulposus, distributing load effectively to maintain IVD structure and function. Their mechanical properties must be tunable to accommodate individual patient needs and various disease stages, thereby reducing symptoms and enhancing patient quality of life.
(4) Enhanced Mechanical Strength with Dynamic Bonds: Future research should focus on enhancing the mechanical strength and self-healing efficiency of hydrogels. Innovations could include modifying the composition and ratios of hydrogel components to improve their biological properties.
(5) In designing therapeutic systems, it is necessary to leverage cutting-edge technologies further, utilizing innovative approaches such as AI-driven intelligent material screening, 4D/5D bioprinting, and organoids to advance the development of personalized medicine and IVDD precision therapy.

## CRediT authorship contribution statement

**Zhengrong Gu:** Visualization, Validation, Supervision. **Yi He:** Investigation, Funding acquisition, Data curation. **Honglin Xiang:** Data curation, Conceptualization. **Qiwei Qin:** Funding acquisition, Formal analysis, Data curation. **Xinna Cao:** Methodology, Investigation. **Ke Jiang:** Validation, Supervision, Software, Resources. **Haoshaqiang Zhang:** Conceptualization, Data curation, Formal analysis. **Yuling Li:** Writing – review & editing, Writing – original draft, Visualization, Validation, Supervision, Conceptualization.

## Ethics approval and consent to participate

This is a review article, the article does not involve experimental studies in animals and humans, so no ethically relevant documents are provided.

## Declaration of competing interest

The authors declare that they have no known competing financial interests or personal relationships that could have appeared to influence the work reported in this paper.

## Data Availability

No data was used for the research described in the article.
